# Effect of heat transfer on hybrid nanofluid flow in converging/diverging channel using fuzzy volume fraction

**DOI:** 10.1038/s41598-022-24259-6

**Published:** 2022-12-02

**Authors:** Lalchand Verma, Ramakanta Meher, Zakia Hammouch, Haci Mehmet Baskonus

**Affiliations:** 1 Department of Mathematics and Humanities, Sardar Vallabhbhai National Institute of Technology Surat, Surat, 395 007 Gujarat India; 2 Division of Applied Mathematics, Thu Dau Mot University, Thu Dau Mot, Binh Duong Province Vietnam; 3 Systems and Sustainable Environment Laboratory, Private University of Fez (UPF), Fez, Morocco; 4 Department of Medical Research, China Medical University Hospital, Taichung, Taiwan; 5 Department of Sciences, Ecole Normale Superieure, Moulay Ismail University, Meknes, Morocco; 6 Department of Mathematics and Science Education, Faculty of Education, Harran University, Sanliurfa, Turkey

**Keywords:** Applied mathematics, Computational science

## Abstract

This work explores the magneto-hydrodynamics (MHD) Jeffery–Hamel nanofluid flow between two rigid non-parallel plane walls with heat transfer by employing hybrid nanoparticles, especially Cu and Cu-Al$_{2}$O$_{3}$. Here the MHD nanofluid flow problem is extended with fuzzy volume fraction and heat transfer with diverse nanoparticles to cover the influence of thermal profiles with hybrid nanoparticles on the fuzzy velocity profiles. The nanoparticle volume fraction is described with a triangular fuzzy number ranging from 0 to $5\%$. A novel double parametric form-based homotopy analysis approach is considered to study the fuzzy velocity and temperature profiles with hybrid nanoparticles in both convergent and divergent channel positions. Finally, the efficiency of the proposed method has been demonstrated by comparing it with the available results in a crisp environment for validation.

## Introduction

In recent years, the research on the fluid flow problem has attracted more and more attention from various researchers and scientists due to its applications in river flow, different technical processes, biology, industry, etc. The incompressible, viscous fluid flow through the convergence/divergence channel is commonly referred to as the Jeffrey Hamel flow^1,2^. Since the pioneering research, many researchers have attempted to expand the flow of divergent and convergent channels by taking into different effects such as MHD and slip and heat transfer phenomena^3–8^.

In recent years nanotechnology has offered a wide range of options for creating colloidal suspensions of various metal particles less than 100 nm in various normal fluids. Choi^9^ was the first to work in this area and coined the name “nanofluid”. Nanofluids, a recently discovered class of fluids, have opened up a new research platform that has proven very valuable in technological and industrial domains. Nanofluids are a different heat transfer fluid than conventional fluids because they have unique heat transfer capabilities. According to researchers, including nanoscale metal particles in classical fluids is thought to give strong heat transmission capabilities. Nanoparticles diluted with many common fluids can significantly alter the flow properties to improve heat transfer.

Hybrid nanofluids are novel types of nanofluid that researchers have recently used in their studies. It is created by scattering two distinct kinds of nanoparticles across the base fluid. These nanofluids boost pressure drop and heat transfer properties by exchanging different solutions. As a result, various investigations on heat transmission in hybrid nanofluid flow have been done. The latest production of hybrid nanofluid and exploration of the preparation process has been analysed by Sadik et al.^10^, and Sarkar et al.^11^. Das^12^ drew the attention of the researchers to hybrid nanofluids, including their production, challenges, and remarkable thermophysical properties. Ghadikolaei et al.^13^ calculated a micropolar dusty fluid’s heat transfer and magnetohydrodynamics flow on a porous medium containing Cu-Al$_{2}$O$_{3}$/H$_{2}$O hybrid nanoparticles. In a hybrid nanofluid flow, Chamkha et al .^14^ examined the heat transmission process in a rotating system between stretchable surfaces. They found that as the injection and radiation parameters are enhanced, the heat transfer rate increases.Waini et al.^15^ investigated the numerical simulation of Cu-Al$_{2}$O$_{3}$/H$_{2}$O hybrid nanofluid transient flow by approaching a curved “stretching/shrinking” surface. They found that as the proportion of copper nanoparticles in the fluid increases, so do the temperature and velocity. Waini et al.^16^ investigated the heat transmission of hybrid nanoparticles in a Cu-Al$_{2}$O$_{3}$/H$_{2}$O fluid flow elicited by permeable stretch and shrink plates and showed that the suction parameter raises the skin friction coefficient. Similarly, Sun et al.^17^ performed a numerical and experimental study on heat transfer in a spinning impinging jet in the presence of silver-“multiwall carbon nanotube”/water hybrid nanofluid. Ma et al.^18^ investigated MHD, the convective flow of Ag-MgO/water hybrid nanoparticles in a convergent and divergent channel where as the unsteady flow and heat transfer on “stretching/shrinking” sheets of hybrid nanofluids have been explored by Waini et al .^19^.

Al$_2$O$_3$/water-based forced convection heat transfer and entropy generation analyses were investigated in the rectangular microchannel by^20^with the consideration of three injections. Esfe at al.^21^ studied the experimental and neural networking with rheological characteristics of *MgO* nanoparticles and oils with the base fluid whereas Esfe et al.^22^ examined the thermal conductivity of a hybrid nanofluid made up of SWCNT-MgO particles mixed in ethylene glycol as a base fluid. The heat transfer and entropy generation containing FMWNT/water nanofluids studied in a microchannel in the presence of the magnetic field^23^. Similarly, the nanofluid moving through a microchannel with a triangular corrugated wall, and the effects of magnetic field on heat transfer and entropy generation were investigated^24^.^25^ examined a double-pipe heat exchanger with a sinusoidal-wavy wall, and also studied the effect of the magnetic field that increases the efficiency and reduces the entropy formation. Graphene nanoplatelets and water in a sinusoidal microchannel were used by^26^ to study how discrete heat sources affect the heat transmission and the pressure drop. Heat transmission in functionalized multi-walled nanotubes and water nanofluid is investigated by^27^ in relation to the impacts of adding trapezoidal ribs to microchannels. Similarly,^28^ numerically investigated and utilized the FVM of the convection heat transport, radiation, and entropy generation (EGE) of nanofluids (NFs) in a two- and three-dimensional square enclosure. To the impact, the research of using water/graphene oxide nanofluid flow on heat transfer and the friction coefficient in a circular profile copper tube, an isotherm heat transfer system has been invented and constructed by^29^. Similarly, the impact of various wall shapes on the thermal-hydraulic properties of different channels filled with water-based graphite-SiO2 hybrid nanofluid are examined by^30^.

Jeffery Hamel’s MHD flow and heat transfer in nanofluids is also a topic of interest for many academicians/researchers. The heat transmission of nanofluid flow between non-parallel plates was examined by Sari et al.^31^. Cu nanoparticles in aqueous liquids have also been found to increase the heat transmission between non-parallel plane walls, with the Nusselt number that increases with the nanoparticle volume fraction. Patel and Meher^32^ employed ADM to investigate MHD Jeffery–Hamel nanofluid flow in a channel and observed the variations of the velocity profiles for divergent and convergent channels with several characteristics such as angle, “Hartmann and Reynolds numbers,” . Similarly, Meher and Patel^33–35^ used DTM to explored the heat transfer with MHD Jeffery–Hamel Cu/water nanofluid flow between two unparallel walls. They discovered that as the volume fraction of nanoparticles grows, so does the fluid’s velocity profile in the channel. Khan et al .^36^ investigated the heat transfer of a Cu-Al$_{2}$O$_{3}$/H$_{2}$O hybrid nanofluid flow in a channel using numerical methods based on the Ranga Kutta method. They found that increasing the volume fraction of nanoparticles reduces the fluid’s velocity profile in the divergent channel but raises it in the convergence channel. The heat transfer rate falls in the convergent channel as the nanoparticle volume fraction increases, while it increases in the divergent channel. Similarly, Hafeez et al .^37^ studied the Jeffery–Hamel flow of copper and Graphite’s oxide/water hybrid nanofluid in the channel using a numerical technique and also talked about the issue of heat transmission. They found that increasing the value of the Reynolds number, it increases the velocity and rate of fluid heat transfer in the convergent channel but not in the divergent channel. The heat transfer of Jeffery–Hamel of Cu/water nanofluid flow in channels with the porous medium was explored by Aninshilo et al.^38^. They noticed that the mix raises the temperature when adding nanoparticles to the fluid. The most influential parameters on vectorial crystal growth of highly oriented vertically aligned carbon nanotubes that adaptive neuro-fuzzy technique has studied^39,40^. The frequency response of a concrete plate equipped with nanoparticles was examined under the effect of water^41^ and found that increasing the volume fraction of nanoparticles increases the frequency. In contrast, the aggregation of the nanoparticles might significantly lower the frequency. Berrehal and Sowmya^42^ Utilized SHAM to study the heat transfer of Copper-Silver/water nanofluid flow in convergent and divergent channels and found that when the volume proportion of nanoparticles grew, the velocity profile reduced, but the fluid’s heat transmission in a channel increased. Verma and Meher^43^ have discussed the heat transfer of Cu/Ag nanofluid with fuzzy volume fractions and observed that the heat transfer decreases with increases in the volume fraction in the convergent channel. Still, heat transfer increases with the volume fraction in the divergent channel.

In the Jeffery Hamel flow problems research, as seen in the literature mentioned above, some researchers used pure water while others used single nanoparticles with base fluid. This work explores the magneto-hydrodynamics (MHD) Jeffery–Hamel nanofluid flow between two rigid non-parallel plane walls with heat transfer by employing hybrid nanoparticles, especially Cu and Cu-Al$_{2}$O$_{3}$. The volume fractions play a crucial role in the study of nanofluid flow with the presence of various nanoparticles suspended with the base fluid as water. Here the MHD nanofluid flow problem is extended with fuzzy volume fraction and heat transfer with diverse nanoparticles like Cu-Al$_{2}$O$_{3}$ to study the influence of thermal profiles with hybrid nanoparticles on the fuzzy velocity profiles. The nanoparticle volume fraction is described with a triangular fuzzy number ranging from $0\%$ to $5\%$. A novel double parametric form-based homotopy analysis approach is considered to study the fuzzy velocity and temperature profiles with hybrid nanoparticles in both convergent and divergent channel positions. Finally, the efficiency of the proposed method has been demonstrated by comparing it with the available results in a crisp environment for validation.

This work has been divided into several sections. The basic concept and preliminaries is discussed in “Preliminaries”. “Problem description” discusses the mathematical formulation of the proposed problem while “Double parametric approach based on problem” and “Solution with HAM” covers the application of double parametric approach with HAM. “Convergence analysis of the problem” discusses the convergence analysis of the problem. Finally, “Result and discussion” discusses the result and discussion, while the conclusion is presented in “Conclusion”.

## Preliminaries

This section discusses the basic concept of fuzzy set theory with some mathematical representations.

### Definition 1.1

A fuzzy set $\tilde{B}$ in $\mathbb {R}$,is a function $C:\mathbb {R}\rightarrow [0,1]$ ,where $\mathbb {R}$ is the set of real numbers and a set of order pairs.It can be written as mathematically^44^ as

$$\begin{aligned} \tilde{B}=\lbrace (s, C(s)):x\in \mathbb {R},C(s)\in [0,1]\rbrace \end{aligned}$$

where C is the membership value.

### Definition 1.2

(*Ref.*^45^) A fuzzy set is said to be fuzzy number if it is piece-wise continuous, normal (i.e.$\exists s_{0}\in \mathbb {R}$ s.t. $C(s_{0})=1$), fuzzy convex set and with a compact support.

Also , the “Triangular fuzzy number” (TFN) can be defined as a fuzzy number that is represented by $\tilde{B}=[a,b,c]$. The membership function of TFN $\tilde{B}=[c_{1},c_{2},c_{3}]$ can be defined as

$$\begin{aligned} C(s)={\left\{ \begin{array}{ll} 0 &{} s\leqslant c_{1} \\ \frac{s-c_{1}}{c_{2}-c_{1}}&{} s\in (c_{1},c_{2}]\\ \frac{c_{3}-s}{c_{3}-c_{2}} &{} s\in (c_{2},c_{3}]\\ 0 &{} s\geqslant c_{3} \end{array}\right. } \end{aligned}$$

In this work we consider the nano-particles of volume fraction as an uncertain parameters and can be represented as a TFN.

### Definition 1.3

The $\alpha$-cut set of a “fuzzy number” ${\tilde{B}}$ can be represented by $[{\tilde{B}}]_\alpha$ and is defined as

$$\begin{aligned} {\tilde{[B]}}_\alpha ={\left\{ \begin{array}{ll} b\in \mathbb {R} : C(b)\geqslant \alpha , if \; \alpha \in (0,1]\\ cl\lbrace b\in \mathbb {R} : C(b)\geqslant 0\rbrace , if \; \alpha =0 \end{array}\right. } \end{aligned}$$

Also we can write the TFN $\tilde{B}=[c_{1},c_{2},c_{3}]$ in an interval form using the concept of $\alpha$-cuts^46^ is $[(c_{2}-c_{1})\alpha +c_{1}, c_{3}-(c_{3}-c_{2})\alpha ]$.

### Definition 1.4

The parametric form of $\alpha$-cuts can be written in an interval form as $L=[\underline{L},\overline{L}]$. The parametric form is expressed as^47^

$$\begin{aligned} L=\delta (\overline{L}-\underline{L})+\underline{L} , \delta \in [0,1]. \end{aligned}$$

This concept is known as a double parametric concept.

## Problem description

**Figure 1:**
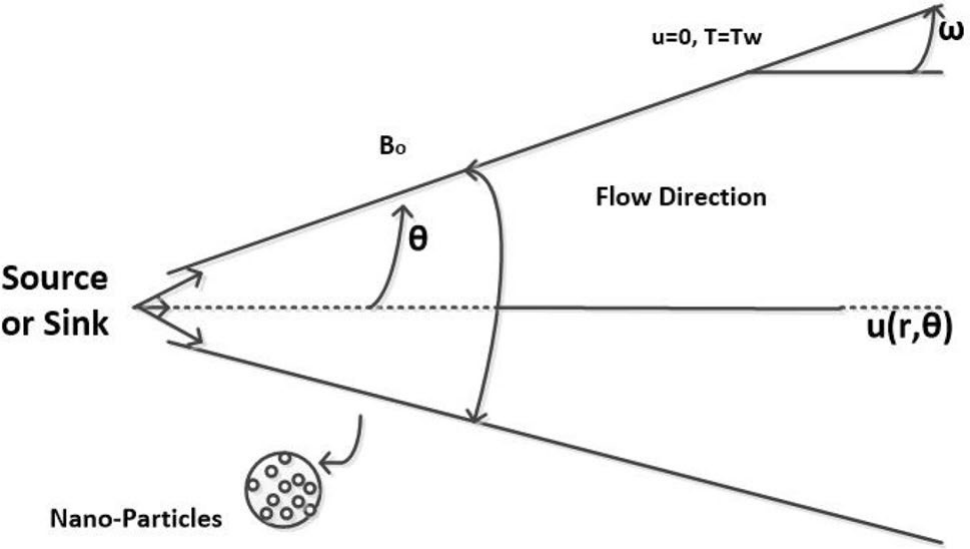
Geometry of the problem.

Consider a two-dimensional laminar viscous incompressible hybrid nanofluid flow between two non-parallel plates from a source or sink. As indicated in Fig. 1, the angle between the duct walls is assumed to be $2\omega$. The physical properties of a nanofluid and effective hybrid nanofluid are represented in Tables 1 and 2, respectively. The flow is supposed to be symmetrical and radial. Here we consider the primary fluid as water, with the presence of hybrid nanoparticles with the base fluid. As previously stated, the velocity field in convection form is in the form $\mathscr {U} (\vartheta (r, \theta ), 0,0)$. The reduced forms of continuity, Navier-Stokes and energy equations^38,42,48^ are

2.1 $$\begin{aligned}{} & {} \frac{\rho _{nf}}{r}\frac{\partial (r\vartheta )}{\partial r}=0 , \end{aligned}$$

2.2 $$\begin{aligned}{} & {} \vartheta \frac{\partial \vartheta }{\partial r}=-\frac{1}{\rho _{nf}}\frac{\partial p}{\partial r}+\mu _{nf}\Big [\frac{\partial ^{2}\vartheta }{\partial r^{2}}+\frac{1}{r}\frac{\partial \vartheta }{\partial r}+\frac{1}{r^{2}}\frac{\partial ^{2}\vartheta }{\partial \theta ^{2}}-\frac{\vartheta }{r^{2}}\Big ]-\frac{\sigma _{nf} B_{0}^{2}}{\rho _{nf}r^{2}}\vartheta , \end{aligned}$$

2.3 $$\begin{aligned}{} & {} \frac{1}{r\rho _{nf}}\frac{\partial p}{\partial \theta }-\frac{2\nu _{nf}}{r^{2}}\frac{\partial (\vartheta )}{\partial \theta }=0, \end{aligned}$$

2.4 $$\begin{aligned}{} & {} \vartheta \frac{\partial T}{\partial r}=\frac{k_{nf}}{(\rho C_{P})_{nf}}\Big [\frac{\partial ^{2}T}{\partial r^{2}}+\frac{1}{r}\frac{\partial T}{\partial r}+\frac{1}{r^{2}}\frac{\partial ^{2}T}{\partial \theta ^{2}}\Big ]+\frac{k_{nf}}{(\rho C_{P})_{nf}}\Big [4\Big \lbrace (\frac{\partial r\vartheta }{\partial r})^{2}+\frac{1}{r^{2}}(\frac{\partial r\vartheta }{\partial \theta })^{2}\Big \rbrace \Big ] \end{aligned}$$

with boundary conditions

2.5 $$\begin{aligned} \vartheta{} & {} =U, \; \frac{\partial \vartheta }{\partial \theta }=0, \; \frac{\partial T}{\partial \theta }=0, \; At \; the \; centerline \;of \;channel, \nonumber \\ \vartheta{} & {} =0, \; T=T_{w}, \; At \; the \; body \; of \; channel \end{aligned}$$

**Figure 2:**
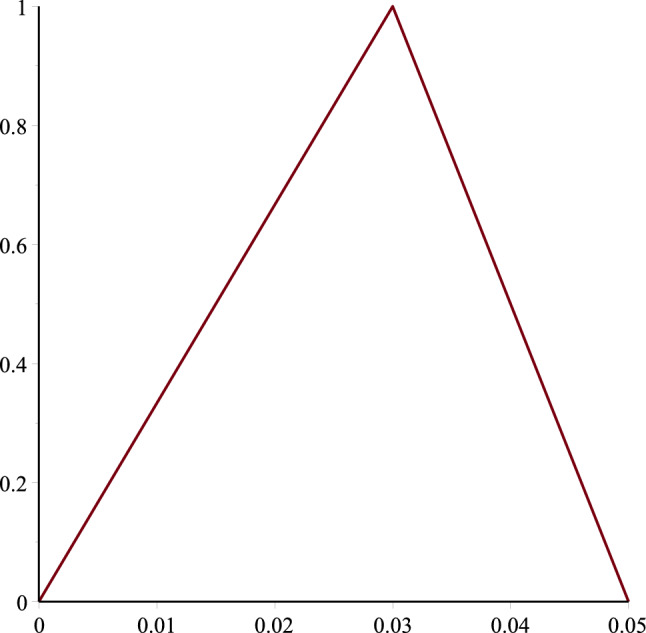
Geometry of TFN $\tilde{\phi }=[0\%,3\%,5\%]$.

**Table 1:** Thermo-physical properties of water and nano-particles^43^.

Thermo-physical properties	Pure water	Cu	Al$_2$O$_3$
Density $$\rho$$ (kg/m$$^{3})$$	997.1	8933	3970
Specific heat $C_{P}$ (J/KgK)	4179	385	765
Thermal conductivity k(W/mk)	0.613	401	40

**Table 2:** The effective models of Hybrid nanofluid of physical parameters^42^.

Thermo-physical properties	Nanofluid	Hybrid nanofluid
Density	$\rho _{nf}=\rho _{f}(1-\phi )+\rho _{s}\phi$	$\rho _{hnf}=[(1-\phi _{2})(1-\phi _{1}+\frac{\rho _{s_{1}}}{\rho _{f}}\phi _{1})+\frac{\rho _{s_{2}}}{\rho _{f}}\phi _{2}]\rho _{f}$
Viscosity	$\mu _{nf} =\frac{\mu _{f}}{(1-\phi )^{2.5}}$	$\mu _{hnf} =\frac{\mu _{f}}{(1-\phi _{1})^{2.5}(1-\phi _{2})^{2.5}}$
Thermal conductivity	$\frac{k_{nf}}{k_{f}}=\frac{(k_{s}+(m-1)k_{f})-(m-1)(k_{f}-k_{s})\phi }{k_{s}+(m-1)k_{f}+(k_{f}-k_{s})\phi }$	$\frac{k_{hnf}}{k_{bf}}=\frac{(k_{s_{2}}+(m-1)k_{bf})-(m-1)(k_{bf}-k_{s_{2}})\phi _{2}}{k_{s_{2}}+(m-1)k_{f}+(k_{bf}-k_{s_{2}})\phi _{2}}$
		Where
		$$\frac{k_{bf}}{k_{f}}=\frac{(k_{s_{1}}+(m-1)k_{f})-(m-1)(k_{f}-k_{s_{1}})\phi _{1}}{k_{s_{1}}+(m-1)k_{f}+(k_{f}-k_{s_{1}})\phi _{1}}$$
Heat capacity	$(\rho C_{P})_{nf}=(1-\phi )(\rho C_{P})_{f}+\phi (\rho C_{P})_{s}$	$(\rho C_{P})_{hnf}$= $(1-\phi _{2})[(1-\phi _{1})(\rho C_{P})_{f}+\phi _{1}(\rho C_{P})_{s_{1}}]+\phi _{2}(\rho C_{P})_{s_{2}}$

Upon considering $\vartheta _{\theta }=0$ for purely radial flow, one can express the velocity profile parameter as

2.6 $$\begin{aligned} f{(\theta )}=r\vartheta (r,\theta ). \end{aligned}$$

Upon assuming the non dimensional form, the non-dimensional degree, velocity, and the temperature parameters can be obtained by dividing that to its maximum value as

2.7 $$\begin{aligned} \zeta =\frac{\theta }{\omega }, \upsilon (\zeta )=\frac{f(\theta )}{f_{max}},\kappa =\frac{T}{T_{w}} \end{aligned}$$

Upon eliminating the pressure p from Eqs. (2.1), (2.2) and with the help of Eqs. (2.8), (2.9), the velocity and temperature profiles can be obtained as

2.8 $$\begin{aligned}{} & {} \upsilon '''(\zeta )+2\omega Re\Lambda _{f}(1-\phi )^{2.5}\upsilon (\zeta )\upsilon '(\zeta )+(4-(1-\phi )^{1.25}Ha)\omega ^{2}\upsilon '(\zeta )=0 \end{aligned}$$

2.9 $$\begin{aligned}{} & {} \frac{k_{nf}}{k_{f}}\kappa ''(\zeta )+\frac{E_{c}P_{r}}{(1-\phi )^{2.5}}\Lambda _{c}[4\omega ^{2}\upsilon ^{2}+(\upsilon ')^{2}]=0 \end{aligned}$$

Here the parameters $\Lambda _{f}$ , $\Lambda _{c}$ and $\Lambda _{k}$ and *Re* can be expressed as

2.10 $$\begin{aligned}{} & {} \Lambda _{f}=(1-\phi )+\frac{\rho _{s}}{\rho _{f}} \phi \end{aligned}$$

2.11 $$\begin{aligned}{} & {} \Lambda _{c}=(1-\phi )+\frac{(\rho C_{P})_{s}}{(\rho C_{P})_{f}} \phi \end{aligned}$$

2.12 $$\begin{aligned}{} & {} \Lambda _{k}=\frac{k_{nf}}{k_{f}}=(\frac{k_{s}+2k_{f})-2(k_{f}-k_{s})\phi }{k_{s}+2k_{f}+\phi (k_{f}-k_{s})}) \end{aligned}$$

2.13 $$\begin{aligned}{} & {} Re=\frac{f_{max}\omega }{\nu _{f}}=\frac{U_{max}r\omega }{\nu _{f}} \end{aligned}$$

2.14 $$\begin{aligned}{} & {} Ha=B_{0}\sqrt{\frac{\sigma }{\rho _{f}\nu _{f}}} \end{aligned}$$

with the following reduced boundary conditions

2.15 $$\begin{aligned} & \upsilon (0) =1, \;\upsilon '(0)=0, \; \kappa '(0)=0,\nonumber \\ & \upsilon (\pm 1) =0, \; \kappa (\pm 1)=1 \end{aligned}$$

The velocity and temperature distribution in practice can be influenced by a small change in the nanofluid’s volume fraction value. So here the volume fraction of the nanoparticles can be treated as an unknown parameter that is stated as a fuzzy number to study the challenges arising in the Jeffery–Hamel fluid flow problems.

Equations (2.10) and (2.11) can be written in an uncertain form as

2.16 $$\begin{aligned}{} & {} \tilde{\upsilon }'''(\zeta )+2\omega Re\tilde{\Lambda }_{f}(1-\tilde{\phi })^{2.5}\tilde{\upsilon }(\zeta )\tilde{\upsilon }'(\zeta )+(4-(1-\tilde{\phi })^{1.25}Ha)\omega ^{2}\tilde{\upsilon '}(\zeta )=0 \end{aligned}$$

2.17 $$\begin{aligned}{} & {} \frac{k_{nf}}{k_{f}}\tilde{\kappa }''(\zeta )+\frac{E_{c}P_{r}}{(1-\tilde{\phi })^{2.5}}\tilde{\Lambda }_{c}[4\omega ^{2}\tilde{\upsilon }^{2}+(\tilde{\upsilon }')^{2}]=0 \end{aligned}$$

subject to the boundary conditions as given by

2.18 $$\begin{aligned} {}& \upsilon (0) =1, \;\upsilon '(0)=0, \; \kappa '(0)=0,\nonumber \\& \upsilon (\pm 1) =0, \; \kappa (\pm 1)=1 \end{aligned}$$

These boundary conditions dictate that the highest values in the velocity profiles can be seen at the centre-line $\zeta =0$ as seen in Fig. 1. Here we have considered the fully developed velocity profile in our discussion. Additionally, the no-slip condition in fluid dynamics asserts that near a solid boundary, the fluid will move with zero velocity to the boundary. It can also be observed that the velocity value is zero at $\zeta =1$ while the fluid velocity at all fluid-solid barriers is equal to that of the solid boundary.

## Double parametric approach based on problem

This section discusses the fuzzy governing model of JHF that has been converted into double parametric form (D.P.F..Here we use the $\alpha$-cut notion to solve the D.P.F. of the fuzzy governing problem of the model. The Eqs. (2.16) and (2.17) can be written in an interval form as:

3.1 $$\begin{aligned}{} & {} \Big \lbrace \underline{\upsilon }'''(\alpha ,\zeta ),\overline{\upsilon }'''(\alpha ,\zeta )\Big \rbrace +2\omega Re \Big [\underline{\Lambda }_{f}(\alpha ),\overline{\Lambda }_{f}(\alpha )\Big ]\Big \lbrace 1-[\underline{\phi }(\alpha ),\overline{\phi }(\alpha )\Big \rbrace ^{2.5}\Big [\underline{\upsilon }'(\alpha ,\zeta ),\overline{\upsilon }'(\alpha ,\zeta )\Big ]\Big [\underline{\upsilon }(\alpha ,\zeta ),\overline{\upsilon }(\alpha ,\zeta )\Big ]\nonumber \\{} & {} \quad + \Big (4-(1-[\underline{\phi }(\alpha ),\overline{\phi }(\alpha )])^{1.25}Ha\Big )\omega ^{2}\Big [\underline{\upsilon }'(\alpha ,\zeta ),\overline{\upsilon }'(\alpha ,\zeta )\Big ]=0 \end{aligned}$$

3.2 $$\begin{aligned}{} & {} \Big \lbrace \underline{\kappa }''(\alpha ,\zeta ),\overline{\kappa }''(\alpha ,\zeta )\Big \rbrace +\frac{E_{c}P_{r}}{\Big (\underline{\Lambda }_{k}(\alpha ),\overline{\Lambda }_{k}(\alpha )\Big )\Big (1-[\underline{\phi }(\alpha ),\overline{\phi }(\alpha )]\Big )^{2.5}}\Big [\underline{\Lambda }_{c}(\alpha ),\overline{\Lambda }_{c}(\alpha )\Big ]\Big [4\omega ^{2}\lbrace (\underline{\upsilon }(\alpha ,\zeta ),\overline{\upsilon }(\alpha ,\zeta )\rbrace ^{2}\nonumber \\{} & {} \quad +\lbrace \underline{\upsilon }'(\alpha ,\zeta ),\overline{\upsilon }'(\alpha ,\zeta )\rbrace ^{2}\Big ]=0 \end{aligned}$$

Upon using another parameter $\delta$ in Eqs. (3.1) and (3.2), we get

3.3 $$\begin{aligned}&\left[\delta (\overline{\upsilon }'''(\alpha ,\zeta )-\underline{\upsilon }'''(\alpha ,\zeta ))+\underline{\upsilon }'''(\alpha ,\zeta )\right]+2\omega Re\left[\left\lbrace \delta (\overline{\Lambda }_{f}(\alpha )-\underline{\Lambda }_{f}(\alpha ))+\underline{\Lambda }_{f}(\alpha )\right\rbrace \left\lbrace 1-\lbrace \delta (\overline{\phi }(\alpha )-\underline{\phi }(\alpha ))\right.\right.\\ &\left.\left.\quad +\underline{\phi }(\alpha )\rbrace \right \rbrace ^{2.5} \left \lbrace \delta (\overline{\upsilon }'(\alpha ,\zeta )-\underline{\upsilon }'(\alpha ,\zeta ))+\underline{\upsilon }'(\alpha ,\zeta )\right \rbrace \left \lbrace \delta (\overline{\upsilon }(\alpha ,\zeta )-\underline{\upsilon }(\alpha ,\zeta ))+\underline{\upsilon }(\alpha ,\zeta )\right \rbrace \right ]\\ &\quad + \left(4-\left(1-[\delta \lbrace \overline{\phi }(\alpha )-\underline{\phi }(\alpha )\rbrace +\underline{\phi }(\alpha )]\right)^{1.25}Ha\right )\omega ^{2}\left\lbrace \delta (\overline{\upsilon }'(\alpha ,\zeta )-\underline{\upsilon }'(\alpha ,\zeta ))+\underline{\upsilon }'(\alpha ,\zeta )\right\rbrace =0 \end{aligned}$$

and

3.4 $$\begin{aligned}&\left[\delta (\overline{\kappa }''(\alpha ,\zeta )-\underline{\kappa }''(\alpha ,\zeta ))+\underline{\kappa }''(\alpha ,\zeta )\right]\\ &\quad +\frac{E_{c}P_{r}}{\left[\delta (\overline{\Lambda }_{k}(\alpha )-\underline{\Lambda }_{k}(\alpha ))+\underline{\Lambda }_{k}(\alpha )\right]\left(1-[\delta (\overline{\phi }(\alpha )-\underline{\phi }(\alpha ))+\underline{\phi }(\alpha )]\right)^{2.5}}\left[\delta (\overline{\Lambda }_{c}(\alpha )-\underline{\Lambda }_{c}(\alpha ))+\underline{\Lambda }_{c}(\alpha )\right]\\ &\quad \times \left[4\omega ^{2}\left\lbrace \delta (\overline{\upsilon }(\alpha ,\zeta )-\underline{\upsilon }(\alpha ,\zeta ))+\underline{\upsilon }(\alpha ,\zeta )\right\rbrace ^{2}+\left\lbrace \delta (\overline{\upsilon }'(\alpha ,\zeta )-\underline{\upsilon }'(\alpha ,\zeta ))+\underline{\upsilon }'(\alpha ,\zeta )\right\rbrace ^{2}\right]=0 \end{aligned}$$

Here $\alpha ,\delta \in [0,1]$ ,$\alpha$ & $\delta$ are the uncertain parameters that control the uncertainty in the given problem . The governing fuzzy model can be expressed as (2.16) and (2.17) written as the D.P.F. so (3.3) and (3.4) .

The double parametric form of different parameters can be expressed as

3.5 $$\begin{aligned}{} & {} \delta (\overline{\upsilon }(\alpha ,\zeta )-\underline{\upsilon }(\alpha ,\zeta ))+\underline{\upsilon }(\alpha ,\zeta )=\upsilon (\alpha ,\delta ,\zeta ) \end{aligned}$$

3.6 $$\begin{aligned}{} & {} \delta (\overline{\upsilon }'(\alpha ,\zeta )-\underline{\upsilon }'(\alpha ,\zeta ))+\underline{\upsilon }'(\alpha ,\zeta )=\upsilon '(\alpha , \delta ,\zeta ) \end{aligned}$$

3.7 $$\begin{aligned}{} & {} \delta (\overline{\upsilon }'''(\alpha ,\zeta )-\underline{\upsilon }'''(\alpha ,\zeta ))+\underline{\upsilon }'''(\alpha ,\zeta )=\upsilon (\alpha ,\delta ,\zeta ) \end{aligned}$$

3.8 $$\begin{aligned}{} & {} \delta (\overline{\Lambda }_{f}(\alpha )-\underline{\Lambda }_{f}(\alpha ))+\underline{\Lambda }_{f}(\alpha ,\zeta )=\Lambda _{f}(\alpha ,\delta ) \end{aligned}$$

3.9 $$\begin{aligned}{} & {} \delta (\overline{\Lambda }_{c}(\alpha )-\underline{\Lambda }_{c}(\alpha ))+\underline{\Lambda }_{c}(\alpha ,\zeta )=\Lambda _{c}(\alpha ,\delta ) \end{aligned}$$

3.10 $$\begin{aligned}{} & {} \delta (\overline{\Lambda }_{k}(\alpha )-\underline{\Lambda }_{k}(\alpha ))+\underline{\Lambda }_{k}(\alpha ,\zeta )=\Lambda _{k}(\alpha ,\delta ) \end{aligned}$$

and

3.11 $$\begin{aligned} \delta (\overline{\phi }(\alpha )-\underline{\phi }(\alpha ))+\underline{\phi }(\alpha ,\zeta )=\phi (\alpha ,\delta ) \end{aligned}$$

Now, from Eqs. (3.3) and (3.4) and using the Eqs. (3.5)–(3.11), it obtain

3.12 $$\begin{aligned} {\upsilon '''}(\alpha ,\delta , \zeta )+2\omega Re\Lambda _{f}(\alpha ,\delta )(1-\phi (\alpha ,\delta ))^{2.5}{\upsilon }(\alpha ,\delta ,\zeta ){\upsilon '}(\alpha ,\delta ,\zeta )+(4-(1-\phi )^{1.25}Ha)\omega ^{2}{\upsilon '}(\alpha ,\delta ,\zeta )=0 \end{aligned}$$

and

3.13 $$\begin{aligned} \kappa ''(\alpha ,\delta ,\zeta )+\frac{E_{c}P_{r}}{\Lambda _{k}(\alpha ,\delta )(1-\phi (\alpha , \delta ))^{2.5}}\Lambda _{c}(\alpha ,\delta )\Big [4\omega ^{2}(\upsilon (\alpha ,\delta , \zeta ))^{2}+(\upsilon '(\alpha ,\delta ,\zeta ))^{2}\Big ]=0 \end{aligned}$$

## Solution with HAM

This section discusses the simulation results of the velocity and temperature profiles of the nano fluid obtained using a homotopy analysis method^43^. The nonlinear operator can be defined as follows:

4.1 $$\begin{aligned} M_{1}[\upsilon (\alpha ,\delta ,\zeta ,q)]&={\upsilon '''}(\alpha ,\delta , \zeta )+2\omega Re\Lambda _{f}(\alpha ,\delta )(1-\phi (\alpha ,\delta ))^{2.5}{\upsilon }(\alpha ,\delta ,\zeta ){\upsilon '}(\alpha ,\delta ,\zeta )\\ {}&\quad +(4-(1-\phi )^{1.25}Ha)\omega ^{2}{\upsilon '}(\alpha ,\delta ,\zeta )\\ M_{2}[\kappa (\alpha ,\delta ,\zeta ,q)]&=\kappa ''(\alpha ,\delta ,\zeta )+\frac{E_{c}P_{r}}{\Lambda _{k}(\alpha ,\delta )(1-\phi (\alpha , \delta ))^{2.5}}\Lambda _{c}(\alpha ,\delta )\Big [4\omega ^{2}(\upsilon (\alpha ,\delta , \zeta ))^{2}+(\upsilon '(\alpha ,\delta ,\zeta ))^{2}\Big ] \end{aligned}$$

and for linear operator , we can write

4.2 $$\begin{aligned} L_{1}[\upsilon (\alpha ,\delta ,\zeta )]=\upsilon '''(\alpha ,\delta , \zeta )\\ L_{2}[\upsilon (\alpha ,\delta ,\zeta )]=\kappa ''(\alpha ,\delta , \zeta ) \end{aligned}$$

with the property

4.3 $$\begin{aligned} L_{1}[d_{1}+d_{2}\zeta +d_{3}\zeta ^{2}]=0\\ L_{2}[b_{1}+b_{2}\zeta ]=0 \end{aligned}$$

Here, $d_{1}, d_{2},d_{3}, b_{1} \; \& \;b_{2}$ are integral constant and $L_{1}, L_{2}$ are the linear operators. Following the solution expression and initial condition rule, it selects a first initial guess as

4.4 $$\begin{aligned}{} & {} \upsilon _{0}(\alpha ,\delta ,\zeta )=1-\zeta ^{2} \nonumber \\{} & {} \kappa _{0}(\alpha ,\delta ,\zeta )=1 \end{aligned}$$

The n-th order deformation equation for $n\geqslant 1$ can be expressed as

4.5 $$\begin{aligned} L_{1}[\upsilon _{n}(\alpha ,\delta ,\zeta )-\chi \upsilon _{n-1}(\alpha ,\delta ,\zeta )]=h_{\upsilon }\mathfrak {R}_{n}(\overrightarrow{\upsilon })_{n-1}(\alpha ,\delta ,\zeta )\\ L_{2}[\kappa _{n}(\alpha ,\delta ,\zeta )-\chi \kappa _{n-1}(\alpha ,\delta ,\zeta )]=h_{\kappa }\mathscr {R}_{n}(\overrightarrow{\kappa })_{n-1}(\alpha ,\delta ,\zeta ) \end{aligned}$$

and

4.6 $$\begin{aligned} \upsilon _{n}(0){} & {} =\upsilon '_{n}(0)=\upsilon _{n}(1)=0\nonumber \\ \kappa '_{n}(0){} & {} =0, \; \kappa _{n}(1)=0 \end{aligned}$$

Upon taking the inverse operator of Eq. (4.5), we get

4.7 $$\begin{aligned} \upsilon _{n}(\alpha ,\delta ,\zeta ){} & {} =\chi _{n}\upsilon _{n-1}(\alpha ,\delta ,\zeta )+h\int _{0}^{\zeta }\int _{0}^{\zeta }\int _{0}^{\zeta }H(\zeta )\mathfrak {R}_{n}(\overrightarrow{\upsilon _{n-1}})d\zeta d\zeta d\zeta +d_{1}+d_{2}\zeta +d_{3}\zeta ^{2}\nonumber \\ \kappa _{n}(\alpha ,\delta ,\zeta ){} & {} =\chi _{n}\kappa _{n-1}(\alpha ,\delta ,\zeta )+h\int _{0}^{\zeta }\int _{0}^{\zeta }H(\zeta )\mathscr {R}_{n}(\overrightarrow{\upsilon _{n-1}})d\zeta d\zeta +b_{1}+b_{2}\zeta \end{aligned}$$

where $\mathfrak {R}_{n}(\overrightarrow{\upsilon })_{n-1}$ and $\mathscr {R}_{n}(\overrightarrow{\kappa })_{n-1}$ are given as

4.8 $$\begin{aligned} \mathfrak {R}_{n}(\overrightarrow{\upsilon }_{n-1}(\alpha ,\delta ,\zeta ))&=\upsilon '''_{n-1}(\alpha ,\delta ,\zeta )\\ {}&\quad + 2\omega Re \Lambda _{f}(\alpha ,\delta )(1-\phi (\alpha ,\delta ))^{2.5}\sum _{i=0}^{n-1}\upsilon _{i}(\alpha ,\delta ,\zeta )\upsilon '_{n-1-i}(\alpha , \delta ,\zeta )\\ {}&\quad +(4-(1-\phi )^{1.25}Ha)\omega ^{2} \upsilon '_{n-1}(\alpha ,\delta ,\zeta )\\ \mathscr {R}_{n}(\overrightarrow{\kappa })_{n-1}(\alpha ,\delta ,\zeta )&=\kappa ''_{n-1}(\alpha ,\delta ,\zeta )+\frac{E_{c}P_{r}}{\Lambda _{k}(\alpha ,\delta )(1-\phi (\alpha , \delta ))^{2.5}}\Lambda _{c}(\alpha ,\delta )\\ {}&\quad \times \sum _{i=0}^{n-1}\Big [4\omega ^{2}\upsilon _{i}(\alpha ,\delta , \zeta )\upsilon _{n-1-i}(\alpha ,\delta , \zeta )+\upsilon '_{i}(\alpha ,\delta ,\zeta )\upsilon '_{n-1-i}(\alpha ,\delta , \zeta )\Big ] \end{aligned}$$

and

4.9 $$\begin{aligned} \chi _{n}={\left\{ \begin{array}{ll} 0 &{} n\leqslant 1\\ 1 &{} n>1 \end{array}\right. } \end{aligned}$$

From Eqs. (4.1) and (4.9), we obtain

4.10 $$\begin{aligned}{} & {} \upsilon _{0}(\zeta ,\alpha ,\delta )=1-\zeta ^{2} \end{aligned}$$

4.11 $$\begin{aligned}{} & {} \upsilon _{1}(\alpha ,\delta ,\zeta )=h\Bigg (\frac{1}{60}S_{1}\zeta ^{2}-\frac{1}{12}(S_{1}+S_{2})\zeta ^{4}\Bigg )+\frac{h}{3}\Bigg (\frac{1}{5}S_{1}+\frac{1}{4}S_{2}\Bigg )\zeta ^{2} \end{aligned}$$

4.12 $$\begin{aligned} \upsilon _{2}(\alpha ,\delta ,\zeta )&=h\Bigg (\frac{1}{60}S_{1}\zeta ^{6}-\frac{1}{12}(S_{1}+S_{2})\zeta ^{4}\Bigg )+h\Bigg (\frac{1}{15}S_{1}+\frac{1}{12}S_{2}\Bigg )\zeta ^{2}+h \Bigg (-\frac{1}{5400}S_{1}^{2}h\zeta ^{10}\nonumber \\{} & {} \quad +\frac{1}{560}(S_{1}^{2}h+S_{1}S_{2}h)\zeta ^{8}+\frac{1}{6}\Bigg (\frac{1}{10}S_{1}h-\frac{3}{100}S_{1}^{2}h-\frac{1}{20}S_{1}S_{2}h-\frac{1}{60}S_{2}^{2}h\Bigg )\zeta ^{6}\Bigg )+h\frac{1}{4}\Bigg (-\frac{1}{3}S_{1}h-\frac{1}{3}S_{2}h\nonumber \\{} & {} \quad +\frac{1}{45}S_{1}^{2}h+\frac{1}{20}S_{1}S_{2}h+\frac{1}{36}S_{2}^{2}h\Bigg )\zeta ^{4}-h\Bigg (\frac{163}{75{,}600}AS_{1}^{2}h+\frac{1}{168}S_{1}S_{2}h-\frac{1}{15}+\frac{1}{240}S_{2}^{2}h-\frac{1}{12}S_{2}h\Bigg )\zeta ^{2} \end{aligned}.$$

Hence

4.13 $$\begin{aligned} \upsilon (\alpha ,\delta ,\zeta )=\upsilon _{0}(\alpha ,\delta ,\zeta )+\upsilon _{1}(\alpha ,\delta ,\zeta )+\upsilon _{2}(\alpha ,\delta ,\zeta )+\cdots \end{aligned}$$

and

4.14 $$\begin{aligned}{} & {} \kappa _{0}(\alpha ,\delta ,\zeta )=1 \end{aligned}$$

4.15 $$\begin{aligned} \kappa _{1}(\alpha ,\delta ,\zeta )&=S_{3}\Bigg (\frac{1}{163{,}800}\omega ^{2}h^{2}S_{1}^{2}+\frac{1}{990}\omega ^{2}h^{2}\Bigg (-\frac{1}{12}S_{1}-\frac{1}{12}S_{2}\Bigg )S_{1}+\frac{1}{13{,}200}h^{2}S_{1}^{2}\nonumber \\{} & {} \quad +\frac{2}{45}\omega ^{2}\Bigg (\Bigg (-\frac{3}{30}+\frac{1}{30}h\Bigg (\frac{1}{15}S_{1}+\frac{1}{12}S_{2}\Bigg )hS_{1}\Bigg )+h^{2}\Bigg (-\frac{1}{12}S_{1}-\frac{1}{12}S_{2}\Bigg )^{2}\Bigg )+\frac{1}{450}h^{2}\Bigg (-\frac{1}{3}S_{1}-\frac{1}{3}S_{2}\Bigg )S_{1}\nonumber \\{} & {} \quad +\frac{1}{14}\omega ^{2}\Bigg (\frac{1}{30}hS_{1}+\Bigg (-2+2h\Bigg (\frac{1}{15}S_{1}+\frac{1}{12}S_{2}\Bigg )\Bigg )h\Bigg (-\frac{1}{12}S_{1}-\frac{1}{12}S_{2}\Bigg )\Bigg )+\Bigg (-\frac{1}{40}+\frac{1}{40}h\Bigg (\frac{1}{15}S_{1}+\frac{1}{12}S_{2}\Bigg )\Bigg )hS_{1}\nonumber \\{} & {} \quad +\frac{1}{56}h^{2}\Bigg (-\frac{1}{3}S_{1}-\frac{1}{3}S_{2}\Bigg )^{2}+\frac{2}{15}\omega ^{2}\Bigg (2h\Bigg (-\frac{1}{12}S_{1}-\frac{1}{12}S_{2}\Bigg )+\Bigg (-1+h(\frac{1}{15}S_{1}+\frac{1}{12}S_{2}\Bigg )\Bigg )^{2}\Bigg )+\cdots \Bigg ) \end{aligned}$$

Hence

4.16 $$\begin{aligned} \kappa (\alpha ,\delta ,\zeta )=\kappa _{0}(\alpha ,\delta ,\zeta )+\kappa _{1}(\alpha ,\delta ,\zeta )+\kappa _{2}+\kappa _{3}(\alpha ,\delta ,\zeta )+ \cdots \end{aligned}$$

Here $$S_{1}=2\omega Re \Lambda _{f}(\alpha ,\delta )(1-\phi (\alpha ,\delta ))^{2.5}$$ and $$S_{2}=(4-(1-\phi (\alpha ,\delta ))^{2.5}Ha)\omega ^{2}$$.

$$\begin{aligned} S_{3}=\frac{E_{c}P_{r}}{\Lambda _{k}(\alpha ,\delta )(1-\phi (\alpha , \delta ))^{2.5}}\Lambda _{c}(\alpha ,\delta ) \end{aligned}$$

## Convergence analysis of the problem

### Theorem 5.1

(*Ref.*^49^) Let $\sum _{n=0}^{\infty }\upsilon _{n}(\alpha ,\delta ,\zeta )$ and $\sum _{n=0}^{\infty }\kappa _{n}(\alpha ,\delta ,\zeta )$ are convergent series that uniformly converges to $\upsilon (\alpha ,\delta ,\zeta )$ and $\kappa (\alpha ,\delta ,\zeta )$, receptively, where $\upsilon (\alpha ,\delta ,\zeta )\in L(R^{+})$ and $\kappa (\alpha ,\delta ,\zeta )\in L(R^{+})$ are generated by an n-th order deformation Eq. (4.5). Then $\upsilon (\alpha ,\delta ,\zeta )$ and $\kappa (\alpha ,\delta ,\zeta )$ are the exact solution of the Eqs. (3.12)and (3.13).

### Proof

Let us express the series of the system as

5.1 $$\begin{aligned} \upsilon (\alpha ,\delta ,\zeta )=\upsilon _{0}(\alpha ,\delta ,\zeta )+\sum _{n=1}^{\infty }\upsilon _{n}(\alpha ,\delta ,\zeta )\\ \kappa (\alpha ,\delta ,\zeta )=\kappa _{0}(\alpha ,\delta ,\zeta )+\sum _{n=1}^{\infty }\kappa _{n}(\alpha ,\delta ,\zeta ) \end{aligned}$$

and they are uniformly convergent. Then

5.2 $$\begin{aligned} lim_{n\rightarrow \infty }\upsilon _{n}(\alpha ,\delta ,\zeta )=0\\ lim_{n\rightarrow \infty }\kappa _{n}(\alpha ,\delta ,\zeta )=0 \end{aligned}$$

Now we have

5.3 $$\begin{aligned} \sum _{n=1}^{j}[\upsilon _{n}(\alpha ,\delta ,\zeta )-\chi \upsilon _{n-1}(\alpha ,\delta ,\zeta )]& = \upsilon _{1}(\alpha ,\delta ,\zeta )+(\upsilon _{2}(\alpha ,\delta ,\zeta )-\upsilon _{1}(\alpha ,\delta ,\zeta )) +(\upsilon _{3}(\alpha ,\delta ,\zeta )\\&\quad -\upsilon _{2}(\alpha ,\delta ,\zeta ))+...+(\upsilon _{j}(\alpha ,\delta ,\zeta )-\upsilon _{j-1}(\alpha ,\delta ,\zeta ))\\ \sum _{n=1}^{j}[\kappa _{n}(\alpha ,\delta ,\zeta )-\chi \kappa _{n-1}(\alpha ,\delta ,\zeta )] & = \kappa _{1}(\alpha ,\delta ,\zeta )+(\kappa _{2}(\alpha ,\delta ,\zeta )-\kappa _{1}(\alpha ,\delta ,\zeta )) +(\kappa _{3}(\alpha ,\delta ,\zeta )\\&\quad -\kappa _{2}(\alpha ,\delta ,\zeta ))+...+(\kappa _{j}(\alpha ,\delta ,\zeta )-\kappa _{j-1}(\alpha ,\delta ,\zeta )) \end{aligned}$$

From Eqs. (5.1), (5.2) and (5.3), we get

5.4 $$\begin{aligned} \sum _{n=1}^{\infty }[\upsilon _{n}(\alpha ,\delta ,\zeta )-\chi \upsilon _{n-1}(\alpha ,\delta ,\zeta )]=lim_{n\rightarrow \infty }\upsilon _{n}(\alpha ,\delta ,\zeta )=0\\ \sum _{n=1}^{\infty }[\kappa _{n}(\alpha ,\delta ,\zeta )-\chi \kappa _{n-1}(\alpha ,\delta ,\zeta )]=lim_{n\rightarrow \infty }\kappa _{n}(\alpha ,\delta ,\zeta )=0 \end{aligned}$$

According to the definition of the operator $L_{1}, L_{2}$, we have

5.5 $$\begin{aligned} L_{1}\Bigg (\sum _{n=1}^{\infty }[\upsilon _{n}(\alpha ,\delta ,\zeta )-\chi \upsilon _{n-1}(\alpha ,\delta ,\zeta )]\Bigg )=\sum _{n=1}^{\infty }L_{1}[\upsilon _{n}(\alpha ,\delta ,\zeta )-\chi \upsilon _{n-1}(\alpha ,\delta ,\zeta )]\\ L_{2}\Bigg (\sum _{n=1}^{\infty }[\kappa _{n}(\alpha ,\delta ,\zeta )-\chi \kappa _{n-1}(\alpha ,\delta ,\zeta )]\Bigg )=(\sum _{n=1}^{\infty }L_{2}[\kappa _{n}(\alpha ,\delta ,\zeta )-\chi \kappa _{n-1}(\alpha ,\delta ,\zeta )] \end{aligned}$$

Using Eq. (4.5), we have

5.6 $$\begin{aligned} h_{\upsilon }H\sum _{n=1}^{\infty }\mathfrak {R}_{n}((\overrightarrow{\upsilon })_{n-1}(\alpha ,\delta ,\zeta ),(\overrightarrow{\kappa })_{n-1}(\alpha ,\delta ,\zeta ))=\sum _{n=1}^{\infty }L_{1}[\upsilon _{n}(\alpha ,\delta ,\zeta )-\chi \upsilon _{n-1}(\alpha ,\delta ,\zeta )]=0\\ h_{\upsilon }H\sum _{n=1}^{\infty }\mathscr {R}_{n}((\overrightarrow{\upsilon })_{n-1}(\alpha ,\delta ,\zeta ),(\overrightarrow{\kappa })_{n-1}(\alpha ,\delta ,\zeta ))=\sum _{n=1}^{\infty }L_{2}[\kappa _{n}(\alpha ,\delta ,\zeta )-\chi \kappa _{n-1}(\alpha ,\delta ,\zeta )]=0 \end{aligned}$$

Since $h_{\upsilon },h_{\kappa }, H\ne 0$, we obtain

5.7 $$\begin{aligned} \sum _{n=1}^{\infty }\mathfrak {R}_{n}((\overrightarrow{\upsilon })_{n-1}(\alpha ,\delta ,\zeta ),(\overrightarrow{\kappa })_{n-1}(\alpha ,\delta ,\zeta ))=0\\ \sum _{n=1}^{\infty }\mathscr {R}_{n}((\overrightarrow{\upsilon })_{n-1}(\alpha ,\delta ,\zeta ),(\overrightarrow{\kappa })_{n-1}(\alpha ,\delta ,\zeta ))=0 \end{aligned}$$

From Eqs. (3.12) and (3.13), we get

5.8 $$\begin{aligned} & \sum _{n=1}^{\infty }\mathfrak {R}_{n}((\overrightarrow{\upsilon })_{n-1}(\alpha ,\delta ,\zeta ),(\overrightarrow{\kappa })_{n-1}(\alpha ,\delta ,\zeta ))=\upsilon '''_{n-1}(\alpha ,\delta ,\zeta )\\ {}&\quad +2\omega Re \Lambda _{f}(\alpha ,\delta )(1-\phi (\alpha ,\delta ))^{2.5}\sum _{i=0}^{n-1}\upsilon _{i}(\alpha ,\delta ,\zeta )\upsilon '_{n-1-i}(\alpha , \delta ,\zeta )+(4-(1-\phi )^{1.25}Ha)\omega ^{2} \upsilon '_{n-1}(\alpha ,\delta ,\zeta )\\&\sum _{n=1}^{\infty }\mathscr {R}_{n}((\overrightarrow{\upsilon })_{n-1}(\alpha ,\delta ,\zeta ),(\overrightarrow{\kappa })_{n-1}(\alpha ,\delta ,\zeta ))=\kappa ''_{n-1}(\alpha ,\delta ,\zeta )+\frac{E_{c}P_{r}}{\Lambda _{k}(\alpha ,\delta )(1-\phi (\alpha , \delta ))^{2.5}}(\alpha ,\delta )\\ {}&\quad \times \sum _{i=0}^{n-1}\Big [4\omega ^{2}\upsilon _{i}(\alpha ,\delta , \zeta )\upsilon _{n-1-i}(\alpha ,\delta , \zeta )+\upsilon '_{i}(\alpha ,\delta ,\zeta )\upsilon '_{n-1-i}(\alpha ,\delta , \zeta )\Big ] \end{aligned}$$

Simplifying Eq. (5.8), we get

5.9 $$\begin{aligned}{} & {} {\upsilon '''}(\alpha ,\delta , \zeta )+2\omega Re\Lambda _{f}(\alpha ,\delta )(1-\phi (\alpha ,\delta ))^{2.5}{\upsilon }(\alpha ,\delta ,\zeta ){\upsilon '}(\alpha ,\delta ,\zeta )+(4-(1-\phi )^{1.25}Ha)\omega ^{2}{\upsilon '}(\alpha ,\delta ,\zeta )=0\nonumber \\ &\quad \kappa ''(\alpha ,\delta ,\zeta )+\frac{E_{c}P_{r}}{\Lambda _{k}(\alpha ,\delta )(1-\phi (\alpha , \delta ))^{2.5}}\Lambda _{c}(\alpha ,\delta )\Big [4\omega ^{2}(\upsilon (\alpha ,\delta , \zeta ))^{2}+(\upsilon '(\alpha ,\delta ,\zeta ))^{2}\Big ]=0 \end{aligned}$$

which is the governing equation of the problem. Hence the series $\sum _{n=0}^{\infty }\upsilon _{n}(\alpha ,\delta ,\zeta )$ and $\sum _{n=0}^{\infty }\kappa _{n}(\alpha ,\delta ,\zeta )$ are converges to exact solution of the problem.

## Result and discussion

**Table 3:** Comparison between of numerical^31^ and applied method for converging channel $\upsilon (\alpha ,\delta ,\zeta )$, when $Re=50, Ha=0, \omega =-3^{0}, \alpha =0,\delta =1, h=-1$ for Cu-H$_2$O nanofluid.

Ha=0		HAM			
$\zeta$	Numerical	2nd-term App.	3rd-term App.	4th-term App.	E = |NM-HAM|
$-$ 1	0.0000000000	0.0000000000	0.0000000000	0.0000000000	0.0000000000
$-$ 0.25	0.9568619246	0.9605584994	0.9564524512	0.9568692097	0.0000927149
$-$ 0.5	0.8114573769	0.8202653623	0.8148138442	0.8114982031	0.0000408262
$-$ 0.75	0.5158279952	0.5220192730	0.5156875085	0.5153640346	0.0004639607
0	1.00000000000	1.0000000000	1.0000000000	1.0000000000	0.0000000000
0.75	0.5158279952	0.5220192730	0.5156875085	0.5153640346	0.0004639607
0.5	0.8114573769	0.8202653623	0.8108138442	0.8110982031	0.0000408262
0.25	0.9568619246	0.9605584994	0.9564524512	0.9568692097	0.0000927149
1	0.0000000000	0.0000000000	0.0000000000	0.0000000000	0.0000000000

**Table 4:** Comparsion between of numerical^31^ and applied method of velocity distribution for diverging channel $\upsilon (\alpha ,\delta ,\zeta )$, when $Re=50, Ha=0, \omega =3^{0}, \alpha =0,\delta =1, h=-1$ for Cu-H$_2$O nanofluid.

Ha=0		HAM			
$\zeta$	Numerical	2nd-term App.	3rd-term App.	4th-term App.	E = |NM-HAM|
$-$ 1	0.0000000000	0.0000000000	0.0000000000	0.0000000000	0.0000000000
$-$ 0.75	0.3461789162	0.3525309398	0.3461072234	0.3461278751	0.0000510411
$-$ 0.5	0.6693201741	0.6793919428	0.6698309976	0.6693351126	0.0000149385
$-$ 0.25	0.9097653427	0.9143344085	0.9101854898	0.9097619558	0.0000033869
0	1.0000000000	1.0000000000	1.0000000000	1.0000000000	1.0000000000
0.25	0.9097653427	0.9143344085	0.9101854898	0.9097619558	0.0000033869
0.5	0.6693201741	0.6793919428	0.6698309976	0.6693351126	0.0000149385
0.75	0.3461789162	0.3525309398	0.3461072234	0.3461278751	0.0000510411
1	0.0000000000	0.0000000000	0.0000000000	0.0000000000	0.0000000000

**Table 5:** Velocity profile of the nanofluid in uncertain case for converging and diverging channel when $Re=15, Ha=500, \alpha =0.1, \delta =0.2, \omega =\mp 3^{0}$ for Copper-H$_2$O nanfluid.

$\zeta$	Convergent channel			Divergent channel		
	2nd-term App.	3rd-term App.	4th-term App.	2nd-term App.	3rd-term App.	4th-term App.
0.0	1	1	1	1	1	1
0.1	0.9921874432	0.9919292193	0.9919490237	0.9899890047	0.9899874993	0.9899875334
0.2	0.9684525709	0.9674835900	0.9675554126	0.9599894087	0.9599834132	0.9599835545
0.3	0.9279137946	0.9259603502	0.9260969575	0.9100913651	0.9100778726	0.9100782044
0.4	0.8691351939	0.8661803257	0.8663702355	0.8404117347	0.8403875445	0.8403881601
0.5	0.7901766055	0.7864821601	0.7866939987	0.7510439978	0.7510058442	0.7510068255
0.6	0.6886637493	0.6847260665	0.6849205361	0.6419881282	0.6419339604	0.6419353347
0.7	0.5618783898	0.5583181500	0.5584629453	0.5130604305	0.5129926433	0.5129942996
0.8	0.4068685332	0.4042683736	0.4043499091	0.3637833443	0.3637135591	0.3637151693
0.9	0.2205786611	0.2192964390	0.2193235989	0.1932552101	0.1932070346	0.1932080668
1.0	0	0	0	0	0	0

**Table 6:** Velocity profile of hybrid nanofluid in uncertain case for converging and diverging channel when $Re=15, Ha=500, \alpha =0.1, \delta =0.2, \omega =\mp 3^{0}$ for Cu-Al$_{2}$O$_{3}$/H$_2$O nanfluid.

$\zeta$	Convergent channel			Divergent divergent		
	2nd-term App.	3rd-term App.	4th-term App.	2nd-term App.	3rd-term App.	4th-term App.
0.0	1	1	1	1	1	1
0.1	0.9920493917	0.9918244171	0.9918401878	0.9898488815	0.9898480248	0.9898480266
0.2	0.9679170680	0.9670736613	0.9671306719	0.9594459312	0.9594432208	0.9594432310
0.3	0.9267715600	0.9250740417	0.9251818613	0.9089323366	0.9089284167	0.9089284470
0.4	0.8672605110	0.8646988390	0.8648474249	0.8385099860	0.8385068264	0.8385068835
0.5	0.7875608130	0.7843681943	0.7845318252	0.7483913312	0.7483906932	0.7483907693
0.6	0.6854487989	0.6820600906	0.6822074996	0.6387291959	0.6387308364	0.6387309151
0.7	0.5583904888	0.5553427805	0.5554494164	0.5095265291	0.5095277678	0.5095278399
0.8	0.4036518912	0.4014408392	0.4014980196	0.3605261038	0.3605240941	0.3605241617
0.9	0.2184293587	0.2173480158	0.2173650871	0.1910801612	0.1910761524	0.1910762029
1.0	0	0	0	0	0	0

To find the result, we assume that $\phi$ is a fuzzy number and which is taking a fuzzy triangular number, depicted in Fig. 2, i.e., $[0\%, 3\%, 5\%].$

This research investigates Jeffery- Hamel’s hybrid nanofluid flow and heat transfer in a channel with non-parallel walls and uses HAM to simulate the solution. Here we utilise H$_2$O as the base fluid and Cu and Cu-Al$_{2}$O$_{3}$ as the nanoparticles and hybrid nanoparticles. Throughout the discussion, the Prandtl value is kept fixed at 6.2. Let’s take a closer look at a specific case i.e., $Ha=0, Re=50, \alpha =0, \delta =1, \omega =-3^{0}$ and $Ha=0, Re=50, \alpha =0, \delta =1, \omega =3^{0}$, respectively, for convergent and divergent channels. Tables 3 and 4 show the numerical result of HAM for the convergent and divergent channels while Tables 5 and 6 has been incorporated in the uncertain case for Cu/H$_2$O and Cu-Al$_{2}$O$_{3}$/H$_{2}$O for different values of the parameters, i.e. $\alpha \ne 0, \delta \ne 0$, using $Ha=500, Re=15, \alpha =0.1, \delta =0.2, \omega =\mp 3^{0}$, respectively. These tables demonstrate that HAM is a reliable and accurate method for solving Jeffery–Hamel flow problems in both convergent and divergent channels, and the results are in good accord^31^ in the crisp situation. The error approximation has been introduced with: Error=$|NM-HAM|$. Finally, the effects of the different parameters such as “Reynolds number”, “Inclination of angle”, “Hartman’s number”, “Eckert number”, and the effect of volume fraction have been done on the fluid’s velocity profile and fluid temperature in the Cu/H$_2$O and Cu-Al$_2$O$_3$/H$_2$O nanofluid for the convergent and divergent channel.

### Effect of inclination angle

**Figure 3:**
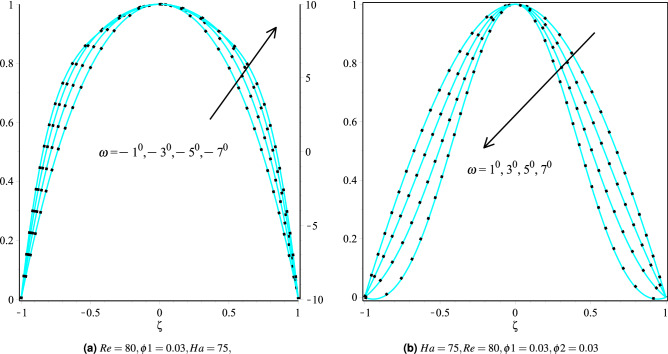
Velocity distribution of the fluid in converging and diverging channel with particular angle for Cu and Cu-Al$_{2}$O$_{3}$/H$_{2}$O, respectively.

**Figure 4:**
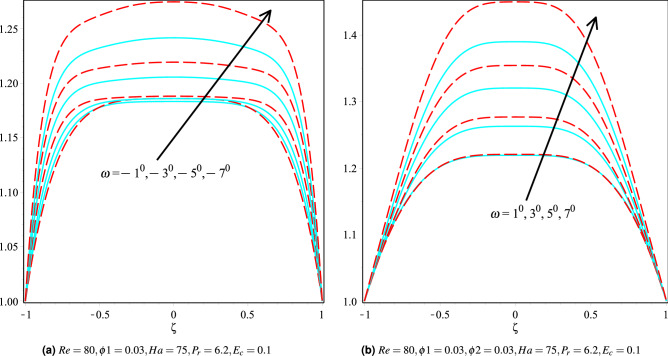
The heat boundary layers distribution in converging and diverging channel with particular $\omega$ for Cu/H$_2$O and Cu-Al$_{2}$O$_{3}$/H$_{2}$O.

It can be noticed that the inclination angle with nanoparticles Cu/H$_2$O and Cu-Al$_{2}$O$_{3}$/H$_{2}$O corresponds to the convergent and divergent channels affects the velocity profiles. Figure 3a,b show that the thickness of the boundary layer for convergent and divergent channels decreases as the inclination angle $\omega$ increases. Physically, when the impact of the wall on the flow of fluid lessens as $\omega$ grows, the velocity of the stream decreases in diverging circumstances. The variations of the fluid temperature for Cu/H$_2$O and Cu-Al$_{2}$O$_{3}$/H$_{2}$O is shown for both channels in Fig. 4a,b. It can be observed that In both cases, i.e., Cu/H$_2$O and Cu-Al$_{2}$O$_{3}$/H$_{2}$O, the temperature of the fluid be increased in both channels, i.e., $\omega <0$ (convergent channel) and $\omega >0$ (divergent channel). It can also be noted that the velocity profiles for Cu/H$_2$O and Cu-Al$_{2}$O$_{3}$/H$_{2}$O nanofluids are identical in both channels, i.e., $\omega >0$ and $\omega <0$ channels. However, the hybrid nanofluid Cu-Al$_{2}$O$_{3}$/H$_{2}$O nanofluid has a higher temperature profile in both channels than the Cu/H$_2$O nanofluid.

### Effect of Reynolds number

**Figure 5:**
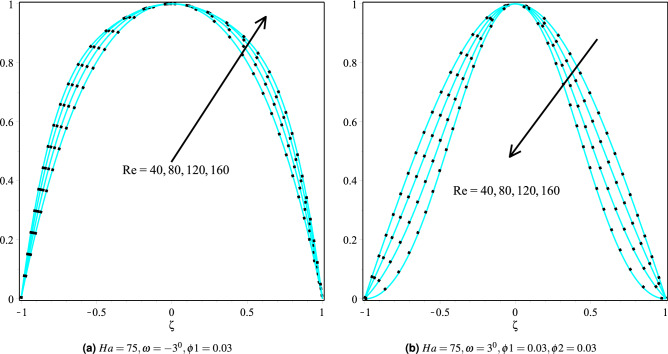
Velocity distribution of the fluid in converging and diverging channel with different Reynolds number for Copper-H$_2$O and Cu-Al$_{2}$O$_{3}$/H$_{2}$O, respectively.

**Figure 6:**
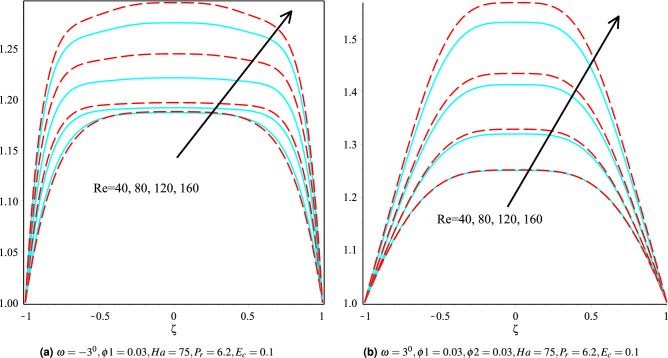
The heat boundary layers distribution in converging and diverging channel with particular Re for Cu/H$_2$O and Cu-Al$_{2}$O$_{3}$/H$_{2}$O.

Figure 5a,b shows the influence of Reynolds number on fluid velocity for Cu/H$_2$O and Cu-Al$_{2}$O$_{3}$/H$_{2}$O nanofluids in $\omega >0$ and $\omega <0$, respectively. The velocity profile for Cu/H$_2$O nanofluid in $\omega <0$ increases as the Reynolds number increases (see Fig. 5a). Since the Reynolds number is the ratio of inertial and viscous forces in physics, as a result, both the momentum barrier layer and fluid velocity are also improved. Figure 5b discusses how the speed of the fluid in the diverging channel decreases as the Reynolds number increases. Similarly, the effect of Reynolds number temperature on the convergent and divergent channels is depicted in Fig. 6a,b. The temperature profile in the $\omega >0$ and $\omega <0$ channels increases as the Reynolds number increases.

### Effect of volume fraction

**Figure 7:**
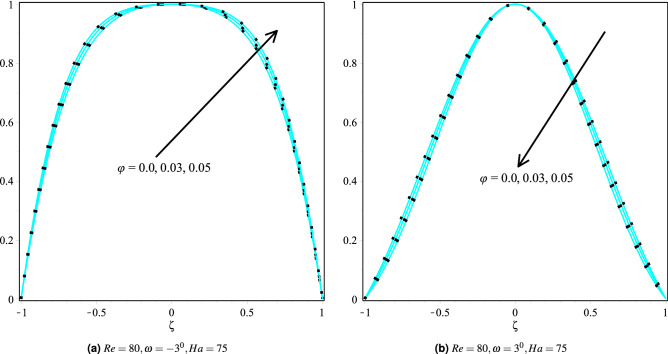
Velocity distribution of the fluid in converging and diverging channel with various $\phi$ for Copper-H$_2$O and Cu-Al$_{2}$O$_{3}$/H$_{2}$O.

**Figure 8:**
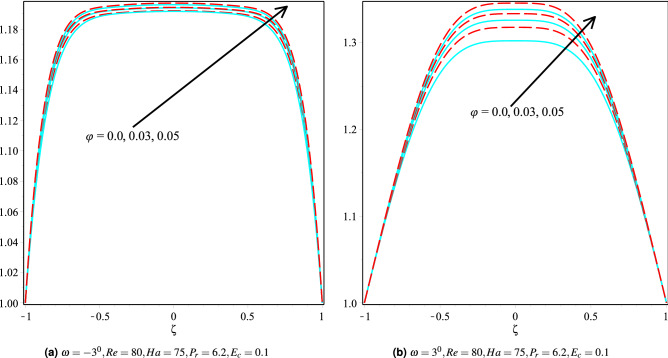
The heat boundary layers distribution in converging and diverging channel with various $\phi$ for Cu/H$_2$O and Cu-Al$_{2}$O$_{3}$/H$_{2}$O.

Figure 7a,b discusses the velocity profiles of Cu/H$_2$O and Cu-Al$_{2}$O$_{3}$/H$_{2}$O nanofluids in $\omega >0$ and $\omega <0$ channels for different values of volume fraction and with other constant parameters. It can be seen that the fluid’s velocity profile increases in convergent channels as the volume fraction increases, but the converse is true in the case of divergent channels. The increased velocity upon introducing the nanoparticles is due to nanoparticles’ fantastic ability to move swiftly inside the fluid. The influence of nanoparticles on the fluid velocity in the convergent and divergent channels for Cu/H$_2$O and Cu-Al$_2$O$_3$/H$_2$O nanofluids are shown in Fig. 8a,b. It can also be seen that the velocity profiles for Cu/H$_2$O and Cu-Al$_{2}$O$_{3}$/H$_{2}$O nanofluid are similar in both the channels, i.e., $\omega >0$ and $\omega <0$ channels.Whereas the fluid temperature in Cu/H$_2$O and Cu-Al$_{2}$O$_{3}$/H$_{2}$O nanofluid is almost identical in the convergent channel. Still, the fluid temperature in Cu-Al$_{2}$O$_{3}$/H$_{2}$O is greater than the Cu/H$_2$O nanofluid.

### Effect of Hartmann number

**Figure 9:**
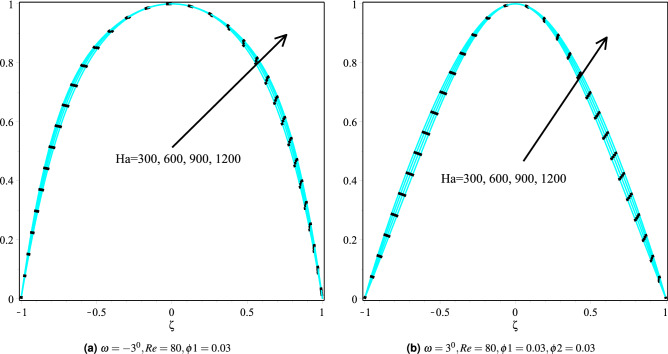
Velocity distribution of the fluid in converging and diverging channel with various Hartmann number for Copper-H$_2$O and Cu-Al$_{2}$O$_{3}$/H$_{2}$O.

**Figure 10:**
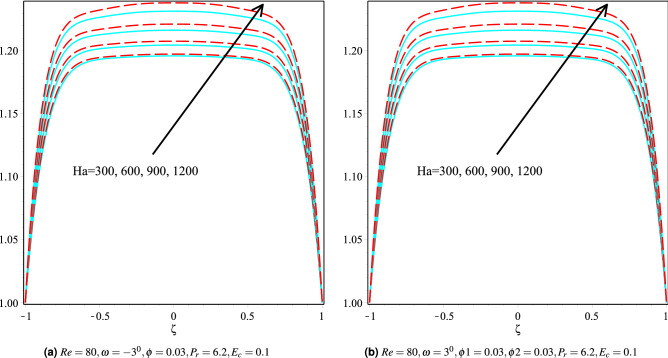
The heat boundary layers distribution in converging and diverging channel with various Ha for Cu/H$_2$O and Cu-Al$_{2}$O$_{3}$/H$_{2}$O.

Figure 9a,b depicts the effect of the Hartmann number on the fluid velocity for Cu and Cu-Al$_{2}$O$_{3}$/H$_{2}$O nanoparticles in $\omega >0$ and $\omega <0$ channels. It can be seen that upon increasing the Hartmann number raises the velocity of the fluid for both $\omega >0$ and $\omega <0$ channels. While Fig. 10a,b depicts the variation in fluid temperature for both channels with different values of Hartmann number and keeping other parameters fixed. It can also be observed that a more significant Harmann number raises the fluid temperature for both $\omega >0$ and $\omega <0$ channels. Physically, the dominant magnetic field increases the viscous forces, resulting in increased resistance and, as a result, increases the heat production in the fluid. As a result, in both the $omega> 0$ and $omega <0$ channels, the fluid temperature rises. It’s also worth noting that the velocity profiles on both channels are also nearly identical. Cu / H$_2$O and Cu-Al$_{2}$O$_{3}$/H$_{2}$O nanofluids have $omega> 0$ and $omega <0$ channels, respectively, although the hybrid nanofluid Cu-Al$_{2}$O$_{3}$/H$_{2}$O has a greater temperature profile in both channels.

### Effect of Eckert number

**Figure 11:**
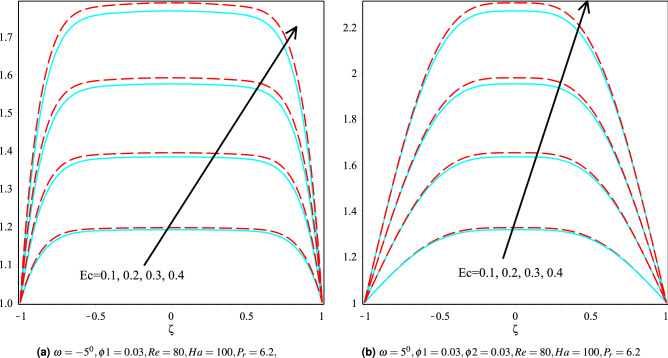
The heat boundary layers distribution in converging and diverging channel with various Eckert number for Cu/H$_2$O and Cu-Al$_{2}$O$_{3}$/H$_{2}$O, respectively.

Figure 11a,b discusses the fluctuation in the temperature profile for Cu/H$_2$O and Cu-Al$_{2}$O$_{3}$/H$_{2}$O nanofluids for various Eckert numbers keeping other parameters fixed in both convergent and divergent channels. It can be observed that as the Eckert number increases, the temperature profile is observed to increase, and it can also be seen that the Cu-Al$_{2}$O$_{3}$/H$_{2}$O nanofluid fluid temperature is higher than that of the Cu/H$_2$O nanofluid in both the channels.

**Figure 12:**
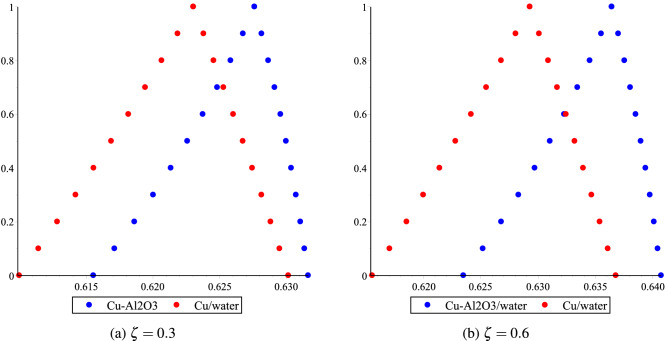
Effect of $\phi =[0\%,3\%,5\%]$ for Cu-H$_2$O and Cu-Al$_{2}$O$_{3}$/H$_{2}$O on fluid velocity distribution bound $\upsilon (\alpha ,\delta ,\zeta )$ in $\omega <0$.

**Figure 13:**
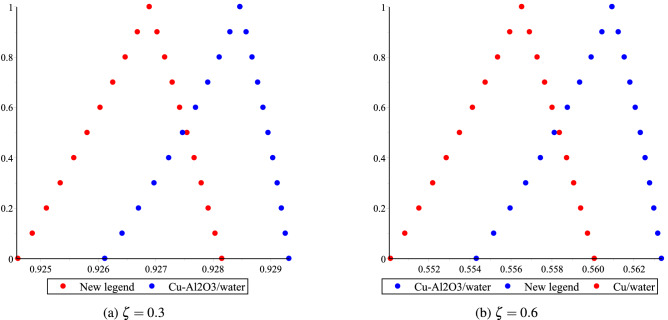
Effect of $\phi =[0\%,3\%,5\%]$ for Cu-H$_2$O and Cu-Al$_{2}$O$_{3}$/H$_{2}$O on fluid velocity distribution bound $\upsilon (\alpha ,\delta ,\zeta )$ in $\omega >0$.

**Figure 14:**
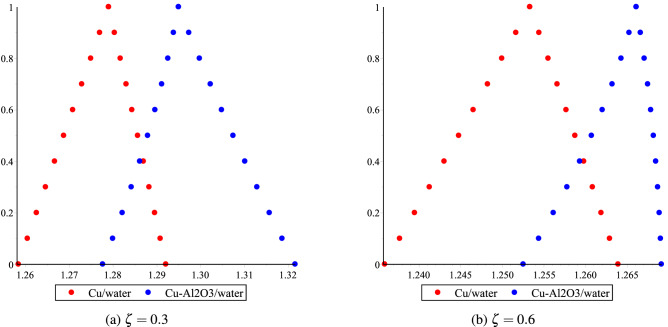
Effect of $\phi =[0\%,3\%,5\%]$ for Cu-H$_2$O and Cu-Al$_{2}$O$_{3}$/H$_{2}$O on fluid temperature distribution bound $\upsilon (\alpha ,\delta ,\zeta )$ in $\omega <0$.

**Figure 15:**
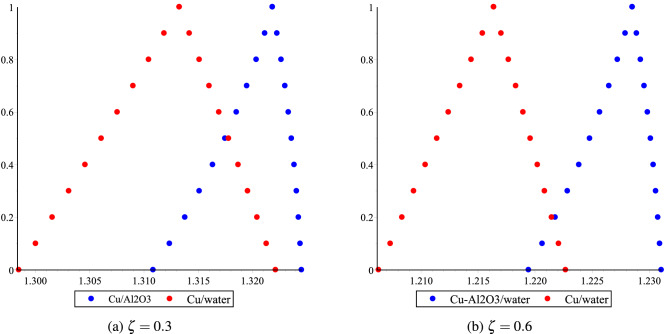
Effect of $\phi =[0\%,3\%,5\%]$ for Cu-H$_2$O and Cu-Al$_{2}$O$_{3}$/H$_{2}$O on fluid temperature distribution bound $\upsilon (\alpha ,\delta ,\zeta )$ in $\omega >0$ with.

The fuzzy velocities and temperatures profiles of Cu/H$_2$O and Cu-Al$_2$O$_3$/H$_2$O have been depicted in Figs. 12a,b, 13a,b, 14a,b and 15a,b by considering volume fraction as a TFN, i.e. $\phi =[0\%, 3\%, 5\%]$ with $Re=80, Ha=75, \omega =-5^{0}$. The graphs depict the fuzzy non-dimensional velocity and temperature profiles for $\zeta =0.3,\; \& \; 0.6$ in both converging and diverging channels. The fuzzy velocities are the TFN shown in the diagram at various channel points. Finally, the velocity bounds of Cu-H$_2$O and Cu-Al$_{2}$O$_{3}$/H$_{2}$O nanofluid have been provided in Tables 7, 8, 9 and 10 for various values of $\alpha$ and $\zeta$ in convergent and divergent channel. The entire table depicts the fluctuation in nanofluid velocity for an unknown volume fraction, i.e., $\phi =[0\%, 3\%, 5\%]$. It’s worth noting that when the volume fraction decreases, it becomes a purely fluid flow problem.

**Table 7:** Fuzzy velocity distribution of lower and upper bound in converging channel when various values of $\zeta$ and $\alpha =0.5 , Ha=500, Re=15, \omega =-3^{0}$.

$\alpha =0.5$	Cu-H$_2$O		Cu-Al$_2$O$_3$/H$_2$O	
$\zeta$	$\underline{\upsilon }(\alpha ,\delta ,\zeta )$	$\overline{\upsilon }(\alpha ,\delta ,\zeta )$	$\underline{\upsilon }(\alpha ,\delta ,\zeta )$	$\overline{\upsilon }(\alpha ,\delta ,\zeta )$
0.0	1	1	1	1
0.1	0.9918292485	0.9920868357	0.9919680644	0.9921665739
0.2	0.9670937322	0.9680962172	0.9676343745	0.9684065373
0.3	0.9251217040	0.9272706193	0.9262821314	0.9279356757
0.4	0.8647886329	0.8683359993	0.8667080766	0.8694331371
0.5	0.7845152207	0.7894925558	0.7872161699	0.7910297593
0.6	0.6822752142	0.6884173291	0.6856215044	0.6903093047
0.7	0.5556228934	0.5622930588	0.5592763469	0.5643388671
0.8	0.4017519152	0.4078802317	0.4051323505	0.4097474921
0.9	0.2175982433	0.2216505630	0.2198541961	0.2228734174
1.0	0	0	0	0

**Table 8:** Fuzzy velocity distribution of lower and upper bound in diverging channel when various values of $\zeta$ and $\alpha =0.5, Ha=500, Re=15, \omega =3^{0}$.

$\alpha =0.5$	Cu-H$_2$O		Cu-Al$_2$O$_3$/H$_2$O	
$\zeta$	$\underline{\upsilon }(\alpha ,\delta ,\zeta )$	$\overline{\upsilon }(\alpha ,\delta ,\zeta )$	$\underline{\upsilon }(\alpha ,\delta ,\zeta )$	$\overline{\upsilon }(\alpha ,\delta ,\zeta )$
0.0	1	1	1	1
0.1	0.9897796094	0.9899657336	0.9896785867	0.9899258572
0.2	0.9591821756	0.9598975373	0.9587941005	0.9597442324
0.3	0.9083868823	0.9098895443	0.9075723987	0.9095673755
0.4	0.8376526561	0.8400665666	0.8363457581	0.8395487170
0.5	0.7472598588	0.7505365186	0.7454883752	0.7498330691
0.6	0.6374307565	0.6413245820	0.6353290209	0.6404879343
0.7	0.5082298729	0.5122891736	0.5060426537	0.5114161819
0.8	0.3594442520	0.3630187751	0.3575216649	0.3622493343
0.9	0.1904414947	0.1927069287	0.1892250595	0.1922188569
1.0	0	0	0	0

**Table 9:** Fuzzy velocity distribution of lower and upper bound in converging channel for various values of $\alpha$ and $\zeta =0.5 , Ha=75, Re=80, \omega =-5^{0}$.

$\zeta =0.5$	Cu-H$_2$O		Cu-Al$_2$O$_3$/H$_2$O	
$\alpha$	$\underline{\upsilon }(\alpha ,\delta ,\zeta )$	$\overline{\upsilon }(\alpha ,\delta ,\zeta )$	$\underline{\upsilon }(\alpha ,\delta ,\zeta )$	$\overline{\upsilon }(\alpha ,\delta ,\zeta )$
0.0	0.8325592687	0.8487777423	0.8385398927	0.8516715629
0.1	0.8337230372	0.8482513479	0.8398353628	0.8514446367
0.2	0.8348610267	0.8477153621	0.8410564152	0.8511964620
0.3	0.8359735463	0.8471697101	0.8422052303	0.8509266162
0.4	0.8370609005	0.8466143167	0.8432839202	0.8506346693
0.5	0.8381233900	0.8460491055	0.8442945310	0.8503201831
0.6	0.8391613101	0.8454739998	0.8452390456	0.8499827111
0.7	0.8401749531	0.8448889223	0.8461193863	0.8496217978
0.8	0.8411646058	0.8442937944	0.8469374168	0.8492369793
0.9	0.8421305518	0.8436885369	0.8476949450	0.8488277824
1.0	0.8430730705	0.8430730705	0.8483937246	0.8483937246

**Table 10:** Fuzzy velocity distribution of lower and upper bound in diverging channel when for various values $\alpha$ and $\zeta =0.5 , Ha=75, Re=80, \omega =5^{0}$.

$\zeta =0.5$	Cu-H$_2$O		Cu-Al$_2$O$_3$/H$_2$O	
$\alpha$	$\underline{\upsilon }(\alpha ,\delta ,\zeta )$	$\overline{\upsilon }(\alpha ,\delta ,\zeta )$	$\underline{\upsilon }(\alpha ,\delta ,\zeta )$	$\overline{\upsilon }(\alpha ,\delta ,\zeta )$
0.0	0.8530016642	0.8773569881	0.8643903774	0.8865233199
0.1	0.8547323687	0.8765565619	0.8664816495	0.8860892677
0.2	0.8564268433	0.8757424388	0.8684617147	0.8856229095
0.3	0.8580854803	0.8749145208	0.8703336545	0.8851236182
0.4	0.8580854803	0.8740727089	0.8721004664	0.8845907564
0.5	0.8612967861	0.8732169036	0.8737650653	0.8840236750
0.6	0.8628502154	0.8723470048	0.8753302869	0.8834217156
0.7	0.8643693288	0.8714629113	0.8767988910	0.8827842067
0.8	0.8658544948	0.8705645211	0.8781735634	0.8821104663
0.9	0.8673060781	0.8696517313	0.8794569181	0.8813997998
1.0	0.8687244387	0.8687244387	0.8806515016	0.8806515016

## Conclusion

In this work, a two-parameter fuzzy Homotopy analysis approach is considered with its convergence analysis and studied the MHD fluid flow problem with hybrid nano-particles with the consideration of volume fractions as an uncertain parameter using a triangular fuzzy number ranging from $[0\%, 3\%, 5\%]$. The effects of the hybrid nano particles on dimensionless fuzzy velocities and temperature profiles in convergent and divergent channels are examined numerically as well as graphically with crisp case “$(\alpha =0, \delta =1)$” and in uncertain case “$\alpha =0.1, \delta =0.2$”. The tables demonstrate that HAM is a reliable and accurate method for resolving Jeffery–Hamel flow problems in convergent and divergent channels. Here the effects of different parameters such as “Reynolds number”, “Inclination of angle”, “Hartman’s number”, “Eckert number”, the volume fraction on the physical treatment of the fluid’s velocity profile and fluid temperature with the Cu/H$_2$O and Cu-Al$_2$O$_3$/H$_2$O nanofluids are demonstrated for both the convergent and divergent channel.

The following observations can be noticed as:

(i) The velocity profiles for Cu/H$_2$O and Cu-Al$_{2}$O$_{3}$/H$_{2}$O nanofluids are identical in both channels, i.e., $\omega >0$ and $\omega <0$ channels. However, the hybrid nanofluid Cu-Al$_{2}$O$_{3}$/H$_{2}$O has a higher temperature profile than the Cu/H$_2$O nanofluid in both the channels.
(ii) The temperature profile increases as the Reynolds number rises in both $\omega >0$ and $\omega <0$ channels.
(iii) The fluid temperature is almost identical in both Cu/H$_2$O and Cu-Al$_{2}$O$_{3}$/H$_{2}$O nanofluid in the convergent channel. Still, the temperature distribution in Cu-Al$_{2}$O$_{3}$/H$_{2}$O is greater than the Cu/H$_2$O nanofluid.
(iv) The velocity profiles Cu / H$_2$O and Cu-Al$_{2}$O$_{3}$/H$_{2}$O nanofluids are almost identical while it has a greater temperature distribution in both channels i.e. $omega> 0$ and $\omega <0$.
(v) As the Eckert number increases, the temperature profile is observed to increase. In both channels, the Cu-Al$_{2}$O$_{3}$/H$_{2}$O nanofluid fluid temperature is higher than that of the Cu/H$_2$O nanofluid.
  Finally, it’s worth noting that the fuzzy velocities at various points along the converging and diverging channels can be expressed as fuzzy triangular numbers. The velocity bounds can also be found in the associated parameter’s fixed value of convergent/divergence channel at various points.

## Data Availability

All data generated or analysed during this study are included in this published article.

## List of symbols

$\vartheta$: Velocity of nanofluid along radial direction
p: Fluid pressure
$$\omega >0$$: Diverging channel
$$\omega <0$$: Converging channel
$\sigma$: Conductivity of he fluid
Re: Reynolds number
Ha: Hartmann number
$$B_{0}$$: Magnetic field
$\omega$: Angle of channel
$$\zeta$$: Dimensionless angle
$$\theta$$: Any angle
$$\rho$$: Density
$\phi$: Nanoparticle volume fraction
$$\mu$$: Dynamic viscosity
$$\nu$$: Kinematic viscosity
$$\nu _{nf}$$: Kinematic viscosity
$$\rho _{nf}$$: Effective density
$\mu _{nf}$: Effective dynamic viscosity
$k_{f}$: Conductivities of the base fluid
$k_{s}$: Conductivities of the nanoparticles
$$(\rho C_{p})_{nf}$$: Heat capacity
$k_{nf}$: Conductivity of base fluid
HAM: Homotopy analysis method
D.P.F.: Double parametric form
JHF: Jeffery–Hamel flow
Cu: Copper
$${\text {Al}}_2{\text {O}}_3$$: Aluminum oxide

## Subscript

nf: Nanofluid
f: Base fluid
s: Nano-solid particles
